# Methyl 2-amino-4-(3-chloro­prop­oxy)-5-methoxy­benzoate

**DOI:** 10.1107/S1600536809011374

**Published:** 2009-04-02

**Authors:** Min Zhang, Ran-zhe Lu, Lu-na Han, Wen-bin Wei, Hai-bo Wang

**Affiliations:** a College of Food Science and Light Industry, Nanjing University of Technology, Xinmofan Road No. 5 Nanjing, Nanjing 210009, People’s Republic of China; b College of Science, Nanjing University of Technology, Xinmofan Road No. 5 Nanjing, Nanjing 210009, People’s Republic of China

## Abstract

The asymmetric unit of the title compound, C_12_H_16_ClNO_4_, contains two crystallographically independent mol­ecules. The benzene rings of the two independent mol­ecules are oriented at a dihedral angle of 88.50 (3)°. Intra­molecular N—H⋯O hydrogen bonds involving the methoxybenzoate carbonyl group in each molecule result in the formation of two planar, six-membered rings, oriented at dihedral angles of 1.39 (3) and 0.68 (3)° with respect to the adjacent benzene rings. In the crystal structure, inter­molecular N—H⋯O hydrogen bonds link the mol­ecules into chains along the *a* axis.

## Related literature

For general background to quinazoline derivatives, see: Knesl *et al.* (2006 ▶). For bond-length data, see: Allen *et al.* (1987 ▶).
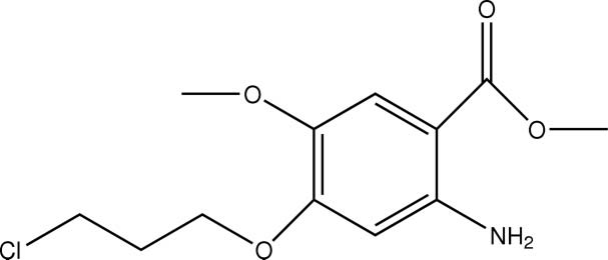


## Experimental

### 

#### Crystal data

C_12_H_16_ClNO_4_
*M* *_r_* = 273.71
Triclinic, 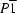

*a* = 8.1080 (16) Å
*b* = 9.818 (2) Å
*c* = 17.739 (3) Å
α = 82.07 (2)°
β = 83.41 (2)°
γ = 89.37 (3)°
*V* = 1389.3 (5) Å^3^
*Z* = 4
Mo *K*α radiation
μ = 0.28 mm^−1^
*T* = 294 K
0.30 × 0.20 × 0.10 mm

#### Data collection

Enraf–Nonius CAD-4 diffractometer
Absorption correction: ψ scan (North *et al.*, 1968 ▶) *T*_min_ = 0.921, *T*_max_ = 0.973
5297 measured reflections
4919 independent reflections
2591 reflections with *I* > 2σ(*I*)
*R*_int_ = 0.041
3 standard reflections frequency: 120 min intensity decay: 1%

#### Refinement

*R*[*F* ^2^ > 2σ(*F* ^2^)] = 0.057
*wR*(*F* ^2^) = 0.157
*S* = 1.01
4919 reflections
325 parameters
H-atom parameters constrained
Δρ_max_ = 0.20 e Å^−3^
Δρ_min_ = −0.22 e Å^−3^

### 

Data collection: *CAD-4 Software* (Enraf–Nonius, 1989 ▶); cell refinement: *CAD-4 Software*; data reduction: *XCAD4* (Harms & Wocadlo, 1995 ▶); program(s) used to solve structure: *SHELXS97* (Sheldrick, 2008 ▶); program(s) used to refine structure: *SHELXL97* (Sheldrick, 2008 ▶); molecular graphics: *ORTEP-3 for Windows* (Farrugia, 1997 ▶) and *PLATON* (Spek, 2009 ▶); software used to prepare material for publication: *SHELXTL* (Sheldrick, 2008 ▶).

## Supplementary Material

Crystal structure: contains datablocks global, I. DOI: 10.1107/S1600536809011374/hk2651sup1.cif

Structure factors: contains datablocks I. DOI: 10.1107/S1600536809011374/hk2651Isup2.hkl

Additional supplementary materials:  crystallographic information; 3D view; checkCIF report

## Figures and Tables

**Table 1:** Hydrogen-bond geometry (Å, °)

*D*—H⋯*A*	*D*—H	H⋯*A*	*D*⋯*A*	*D*—H⋯*A*
N1—H1*A*⋯O3	0.86	2.07	2.709 (4)	131
N1—H1*B*⋯O8^i^	0.86	2.36	3.155 (4)	154
N2—H2*C*⋯O8	0.86	2.09	2.719 (4)	130
N2—H2*C*⋯O8^ii^	0.86	2.43	3.216 (4)	152
N2—H2*D*⋯O3	0.86	2.31	3.119 (4)	156

Symmetry codes: (i) 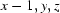; (ii) 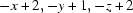.

**Fig. 1.**
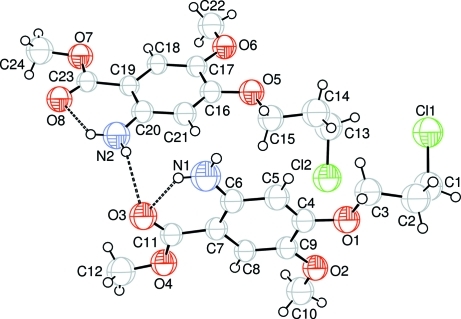
The molecular structure of the title molecule, with the atom-numbering scheme. Hydrogen bonds are shown as dashed lines.

**Fig. 2.**
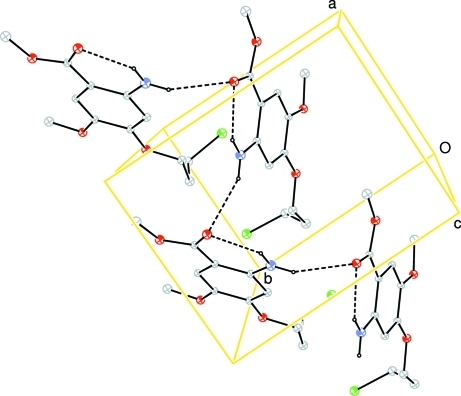
A partial packing diagram of the title compound. Hydrogen bonds are shown as dashed lines. H atoms not involved in hydrogen bonding are omitted.

## supplementary crystallographic information

### Comment

As part of our ongoing studies on quinazoline derivatives (Knesl *et al.*, 2006), we report herein the crystal structure of the title compound.

The asymmetric unit of the title compound contains two crystallographically independent molecules (Fig. 1), in which the bond lengths (Allen *et al.*, 1987) and angles are within normal ranges. Rings A (C4-C9) and A' (C16-C21) are, of course, planar and they are oriented at a dihedral angle of A/A' = 88.50 (3)°. Intramolecular N-H···O hydrogen bonds (Table 1) link the two molecules, also they result in the formations of two six-membered planar rings: B (O3/N1/C6/C7/C11/H1A) and B' (O8/N2/C19/C20/C23/H2C). The dihedral angles between the adjacent rings in each molecule are A/B = 1.39 (3)° and A'/B' = 0.68 (3)°. So, they are also coplanar.

In the crystal structure, intra- and intermolecular N-H···O hydrogen bonds (Table 1) link the molecules into chains along the a axis (Fig. 2), in which they may be effective in the stabilization of the structure.

### Experimental

For the preparation of the title compound, a suspension of methyl 4-(3-chloro- propoxy)-5-methoxy-2-nitrobenzoate (0.016 mol) in HCl (100 ml) was heated at 323-333 K for 5 min, and then a solution of tin(II) chloride (16.0 g, 0.1 mol) in HCl (20 ml) was added dropwise. The reaction mixture was heated at 363-373 K for 45 min. The solid formed was collected and dissolved in water (300 ml). A solution of sodium hydroxide (2N) was added to obtain pH = 8-9. The aqueous solution was then extracted with ethyl acetate (3 × 100 ml). The combined organic layers were dried over magnesium sulfate and concentrated in vacuo to give the title compound (yield; 2.3 g, 51.1%, m.p. 377 K). Crystals suitable for X-ray analysis were obtained by slow evaporation of a methanol solution.

### Refinement

H atoms were positioned geometrically, with N-H = 0.86 Å (for NH_2_) and C-H = 0.93, 0.97 and 0.96 Å for aromatic, methylene and methyl H, respectively, and constrained to ride on their parent atoms, with U_iso_(H) = xU_eq_(C,N), where x = 1.5 for methyl H and x = 1.2 for all other H atoms.

### Crystal data

**Table:**

C_12_H_16_ClNO_4_	*Z* = 4
*M**_r_* = 273.71	*F*(000) = 576
Triclinic, *P*1	*D*_x_ = 1.309 Mg m^−^^3^
Hall symbol: -P 1	Melting point: 377 K
*a* = 8.1080 (16) Å	Mo *K*α radiation, λ = 0.71073 Å
*b* = 9.818 (2) Å	Cell parameters from 25 reflections
*c* = 17.739 (3) Å	θ = 9–13°
α = 82.07 (2)°	µ = 0.28 mm^−^^1^
β = 83.41 (2)°	*T* = 294 K
γ = 89.37 (3)°	Block, colorless
*V* = 1389.3 (5) Å^3^	0.30 × 0.20 × 0.10 mm

### Data collection

**Table:**

Enraf–Nonius CAD-4 diffractometer	2591 reflections with *I* > 2σ(*I*)
Radiation source: fine-focus sealed tube	*R*_int_ = 0.041
graphite	θ_max_ = 25.3°, θ_min_ = 1.2°
ω/2θ scans	*h* = 0→9
Absorption correction: ψ scan (North *et al.*, 1968)	*k* = −11→11
*T*_min_ = 0.921, *T*_max_ = 0.973	*l* = −20→20
5297 measured reflections	3 standard reflections every 120 min
4919 independent reflections	intensity decay: 1%

### Refinement

**Table:**

Refinement on *F*^2^	Primary atom site location: structure-invariant direct methods
Least-squares matrix: full	Secondary atom site location: difference Fourier map
*R*[*F*^2^ > 2σ(*F*^2^)] = 0.057	Hydrogen site location: inferred from neighbouring sites
*wR*(*F*^2^) = 0.157	H-atom parameters constrained
*S* = 1.01	*w* = 1/[σ^2^(*F*_o_^2^) + (0.07*P*)^2^] where *P* = (*F*_o_^2^ + 2*F*_c_^2^)/3
4919 reflections	(Δ/σ)_max_ < 0.001
325 parameters	Δρ_max_ = 0.20 e Å^−^^3^
0 restraints	Δρ_min_ = −0.22 e Å^−^^3^

### Special details

**Table:**

Geometry. All esds (except the esd in the dihedral angle between two l.s. planes) are estimated using the full covariance matrix. The cell esds are taken into account individually in the estimation of esds in distances, angles and torsion angles; correlations between esds in cell parameters are only used when they are defined by crystal symmetry. An approximate (isotropic) treatment of cell esds is used for estimating esds involving l.s. planes.
Refinement. Refinement of F^2^ against ALL reflections. The weighted R-factor wR and goodness of fit S are based on F^2^, conventional R-factors R are based on F, with F set to zero for negative F^2^. The threshold expression of F^2^ > 2sigma(F^2^) is used only for calculating R-factors(gt) etc. and is not relevant to the choice of reflections for refinement. R-factors based on F^2^ are statistically about twice as large as those based on F, and R- factors based on ALL data will be even larger.

### Fractional atomic coordinates and isotropic or equivalent isotropic displacement parameters (Å^2^)

**Table:**

	*x*	*y*	*z*	*U*_iso_*/*U*_eq_	
Cl1	−0.15324 (14)	0.65740 (12)	0.56167 (7)	0.0986 (4)	
Cl2	0.35436 (14)	0.80677 (12)	0.56017 (6)	0.0970 (4)	
O1	−0.0021 (3)	0.9054 (2)	0.70177 (14)	0.0748 (7)	
O2	0.2315 (3)	1.0836 (2)	0.66023 (13)	0.0713 (7)	
O3	0.5434 (3)	0.8244 (3)	0.94591 (14)	0.0784 (7)	
O4	0.6278 (3)	1.0083 (3)	0.85978 (14)	0.0764 (7)	
O5	0.4986 (3)	0.4748 (2)	0.69292 (14)	0.0743 (7)	
O6	0.7348 (3)	0.3139 (2)	0.65050 (13)	0.0713 (7)	
O7	1.1359 (3)	0.2938 (2)	0.84821 (13)	0.0771 (8)	
O8	1.0455 (3)	0.4302 (3)	0.93563 (13)	0.0753 (7)	
N1	0.2572 (4)	0.7010 (3)	0.92623 (18)	0.0916 (10)	
H1A	0.3373	0.6986	0.9542	0.110*	
H1B	0.1770	0.6425	0.9381	0.110*	
N2	0.7544 (4)	0.5646 (3)	0.91837 (16)	0.0812 (10)	
H2C	0.8342	0.5523	0.9466	0.097*	
H2D	0.6729	0.6173	0.9303	0.097*	
C1	−0.1746 (5)	0.8327 (4)	0.5747 (2)	0.0916 (13)	
H1C	−0.0664	0.8770	0.5646	0.110*	
H1D	−0.2447	0.8784	0.5383	0.110*	
C2	−0.2507 (4)	0.8486 (4)	0.6564 (3)	0.0880 (13)	
H2A	−0.2840	0.9436	0.6574	0.106*	
H2B	−0.3503	0.7924	0.6685	0.106*	
C3	−0.1379 (4)	0.8095 (4)	0.7191 (2)	0.0764 (11)	
H3A	−0.1966	0.8168	0.7691	0.092*	
H3B	−0.0983	0.7160	0.7184	0.092*	
C4	0.1200 (4)	0.8952 (3)	0.7498 (2)	0.0599 (9)	
C5	0.1262 (4)	0.8003 (4)	0.8155 (2)	0.0660 (9)	
H5A	0.0414	0.7356	0.8294	0.079*	
C6	0.2574 (4)	0.7991 (3)	0.8619 (2)	0.0591 (9)	
C7	0.3857 (4)	0.8969 (3)	0.84021 (18)	0.0552 (8)	
C8	0.3773 (4)	0.9923 (3)	0.77157 (18)	0.0536 (8)	
H8A	0.4628	1.0561	0.7566	0.064*	
C9	0.2495 (4)	0.9941 (3)	0.72703 (19)	0.0553 (8)	
C10	0.3629 (5)	1.1770 (4)	0.6326 (2)	0.0895 (13)	
H10A	0.3364	1.2330	0.5868	0.134*	
H10B	0.4630	1.1269	0.6213	0.134*	
H10C	0.3786	1.2345	0.6708	0.134*	
C11	0.5223 (4)	0.9032 (3)	0.88748 (19)	0.0562 (8)	
C12	0.7633 (5)	1.0256 (4)	0.9052 (2)	0.0924 (13)	
H12A	0.8318	1.1018	0.8807	0.139*	
H12B	0.8288	0.9434	0.9093	0.139*	
H12C	0.7185	1.0429	0.9554	0.139*	
C13	0.3339 (5)	0.6258 (4)	0.5663 (2)	0.0937 (14)	
H13A	0.2670	0.6044	0.5275	0.112*	
H13B	0.4427	0.5856	0.5564	0.112*	
C14	0.2526 (4)	0.5629 (4)	0.6458 (3)	0.0914 (13)	
H14A	0.1515	0.6130	0.6580	0.110*	
H14B	0.2214	0.4686	0.6434	0.110*	
C15	0.3612 (4)	0.5635 (4)	0.7106 (2)	0.0767 (11)	
H15A	0.4001	0.6559	0.7126	0.092*	
H15B	0.2997	0.5290	0.7595	0.092*	
C16	0.6215 (4)	0.4591 (3)	0.7408 (2)	0.0581 (9)	
C17	0.7518 (4)	0.3703 (3)	0.71711 (18)	0.0546 (8)	
C18	0.8796 (4)	0.3485 (3)	0.76205 (17)	0.0516 (8)	
H18A	0.9653	0.2904	0.7476	0.062*	
C19	0.8872 (4)	0.4108 (3)	0.83011 (17)	0.0485 (7)	
C20	0.7570 (4)	0.4991 (3)	0.85342 (19)	0.0575 (8)	
C21	0.6255 (4)	0.5203 (3)	0.8067 (2)	0.0618 (9)	
H21A	0.5385	0.5774	0.8208	0.074*	
C22	0.8708 (5)	0.2353 (4)	0.6222 (2)	0.0900 (13)	
H22A	0.8456	0.2011	0.5766	0.135*	
H22B	0.8905	0.1595	0.6604	0.135*	
H22C	0.9682	0.2925	0.6104	0.135*	
C23	1.0255 (4)	0.3818 (3)	0.87674 (18)	0.0523 (8)	
C24	1.2755 (5)	0.2593 (4)	0.8929 (2)	0.0937 (14)	
H24A	1.3482	0.1968	0.8683	0.141*	
H24B	1.2343	0.2169	0.9435	0.141*	
H24C	1.3356	0.3416	0.8962	0.141*	

### Atomic displacement parameters (Å^2^)

**Table:**

	*U*^11^	*U*^22^	*U*^33^	*U*^12^	*U*^13^	*U*^23^
Cl1	0.0972 (8)	0.1075 (9)	0.0950 (8)	0.0024 (6)	−0.0306 (6)	−0.0116 (6)
Cl2	0.1009 (8)	0.1059 (9)	0.0894 (8)	0.0022 (6)	−0.0347 (6)	−0.0126 (6)
O1	0.0498 (14)	0.0805 (17)	0.0951 (19)	−0.0060 (12)	−0.0240 (13)	−0.0026 (14)
O2	0.0595 (15)	0.0726 (16)	0.0808 (17)	−0.0087 (12)	−0.0293 (13)	0.0114 (13)
O3	0.0785 (17)	0.0769 (17)	0.0805 (18)	0.0031 (13)	−0.0326 (14)	0.0056 (14)
O4	0.0662 (16)	0.0832 (18)	0.0821 (17)	−0.0119 (14)	−0.0315 (13)	0.0007 (14)
O5	0.0503 (14)	0.0827 (16)	0.0981 (19)	0.0173 (12)	−0.0337 (13)	−0.0217 (14)
O6	0.0619 (15)	0.0820 (17)	0.0800 (17)	0.0160 (12)	−0.0338 (13)	−0.0268 (14)
O7	0.0794 (17)	0.0878 (18)	0.0771 (17)	0.0366 (14)	−0.0420 (14)	−0.0324 (14)
O8	0.0732 (16)	0.0944 (19)	0.0674 (16)	0.0184 (13)	−0.0250 (13)	−0.0315 (14)
N1	0.090 (2)	0.090 (2)	0.089 (2)	−0.0231 (19)	−0.0257 (19)	0.021 (2)
N2	0.083 (2)	0.097 (2)	0.072 (2)	0.0380 (18)	−0.0256 (17)	−0.0295 (18)
C1	0.077 (3)	0.104 (3)	0.092 (3)	−0.018 (2)	−0.038 (2)	0.017 (3)
C2	0.051 (2)	0.087 (3)	0.127 (4)	−0.0035 (19)	−0.026 (2)	−0.006 (3)
C3	0.052 (2)	0.093 (3)	0.086 (3)	−0.016 (2)	−0.0068 (19)	−0.017 (2)
C4	0.0443 (19)	0.067 (2)	0.071 (2)	0.0045 (16)	−0.0116 (17)	−0.0129 (19)
C5	0.059 (2)	0.063 (2)	0.076 (3)	−0.0071 (17)	−0.0097 (19)	−0.0052 (19)
C6	0.055 (2)	0.056 (2)	0.065 (2)	0.0012 (16)	−0.0076 (17)	−0.0035 (17)
C7	0.0506 (19)	0.055 (2)	0.061 (2)	0.0074 (16)	−0.0110 (16)	−0.0096 (17)
C8	0.0398 (17)	0.059 (2)	0.063 (2)	0.0000 (14)	−0.0102 (15)	−0.0059 (17)
C9	0.0496 (19)	0.053 (2)	0.064 (2)	0.0042 (15)	−0.0119 (16)	−0.0044 (16)
C10	0.087 (3)	0.084 (3)	0.092 (3)	−0.022 (2)	−0.033 (2)	0.027 (2)
C11	0.057 (2)	0.055 (2)	0.057 (2)	0.0072 (17)	−0.0102 (17)	−0.0104 (17)
C12	0.075 (3)	0.100 (3)	0.106 (3)	−0.013 (2)	−0.041 (2)	−0.003 (3)
C13	0.089 (3)	0.115 (3)	0.095 (3)	0.047 (3)	−0.051 (3)	−0.045 (3)
C14	0.054 (2)	0.087 (3)	0.140 (4)	0.011 (2)	−0.039 (3)	−0.018 (3)
C15	0.045 (2)	0.091 (3)	0.092 (3)	0.0118 (19)	−0.0162 (19)	0.000 (2)
C16	0.0459 (18)	0.060 (2)	0.071 (2)	0.0022 (15)	−0.0211 (17)	−0.0046 (18)
C17	0.0503 (19)	0.058 (2)	0.059 (2)	0.0051 (15)	−0.0188 (16)	−0.0122 (16)
C18	0.0455 (17)	0.0504 (19)	0.060 (2)	0.0031 (14)	−0.0135 (15)	−0.0041 (16)
C19	0.0481 (18)	0.0469 (18)	0.0492 (19)	0.0014 (14)	−0.0075 (14)	−0.0008 (14)
C20	0.060 (2)	0.057 (2)	0.055 (2)	0.0044 (16)	−0.0116 (17)	−0.0024 (16)
C21	0.0463 (19)	0.065 (2)	0.073 (2)	0.0118 (16)	−0.0070 (17)	−0.0062 (18)
C22	0.094 (3)	0.103 (3)	0.089 (3)	0.031 (2)	−0.046 (2)	−0.043 (2)
C23	0.057 (2)	0.0484 (19)	0.053 (2)	0.0024 (15)	−0.0117 (16)	−0.0073 (16)
C24	0.087 (3)	0.109 (3)	0.099 (3)	0.049 (2)	−0.053 (2)	−0.031 (3)

### Geometric parameters (Å, °)

**Table:**

Cl1—C1	1.772 (4)	C6—C7	1.407 (4)
Cl2—C13	1.773 (4)	C7—C8	1.438 (4)
O1—C4	1.373 (4)	C7—C11	1.471 (4)
O1—C3	1.434 (4)	C8—C9	1.372 (4)
O2—C9	1.395 (4)	C8—H8A	0.9300
O2—C10	1.412 (4)	C10—H10A	0.9600
O3—C11	1.232 (4)	C10—H10B	0.9600
O4—C11	1.350 (4)	C10—H10C	0.9600
O4—C12	1.459 (4)	C12—H12A	0.9600
O5—C16	1.376 (3)	C12—H12B	0.9600
O5—C15	1.439 (4)	C12—H12C	0.9600
O6—C17	1.394 (3)	C13—C14	1.536 (6)
O6—C22	1.423 (4)	C13—H13A	0.9700
O7—C23	1.342 (4)	C13—H13B	0.9700
O7—C24	1.466 (4)	C14—C15	1.527 (5)
O8—C23	1.233 (3)	C14—H14A	0.9700
N1—C6	1.388 (4)	C14—H14B	0.9700
N1—H1A	0.8600	C15—H15A	0.9700
N1—H1B	0.8600	C15—H15B	0.9700
N2—C20	1.392 (4)	C16—C21	1.389 (4)
N2—H2C	0.8600	C16—C17	1.425 (4)
N2—H2D	0.8600	C17—C18	1.376 (4)
C1—C2	1.537 (5)	C18—C19	1.434 (4)
C1—H1C	0.9700	C18—H18A	0.9300
C1—H1D	0.9700	C19—C20	1.420 (4)
C2—C3	1.525 (5)	C19—C23	1.471 (4)
C2—H2A	0.9700	C20—C21	1.420 (4)
C2—H2B	0.9700	C21—H21A	0.9300
C3—H3A	0.9700	C22—H22A	0.9600
C3—H3B	0.9700	C22—H22B	0.9600
C4—C5	1.393 (5)	C22—H22C	0.9600
C4—C9	1.423 (4)	C24—H24A	0.9600
C5—C6	1.417 (4)	C24—H24B	0.9600
C5—H5A	0.9300	C24—H24C	0.9600
			
C4—O1—C3	118.2 (3)	O4—C12—H12A	109.5
C9—O2—C10	117.0 (2)	O4—C12—H12B	109.5
C11—O4—C12	115.7 (3)	H12A—C12—H12B	109.5
C16—O5—C15	118.5 (3)	O4—C12—H12C	109.5
C17—O6—C22	116.5 (2)	H12A—C12—H12C	109.5
C23—O7—C24	115.1 (3)	H12B—C12—H12C	109.5
C6—N1—H1A	120.0	C14—C13—Cl2	111.2 (3)
C6—N1—H1B	120.0	C14—C13—H13A	109.4
H1A—N1—H1B	120.0	Cl2—C13—H13A	109.4
C20—N2—H2C	120.0	C14—C13—H13B	109.4
C20—N2—H2D	120.0	Cl2—C13—H13B	109.4
H2C—N2—H2D	120.0	H13A—C13—H13B	108.0
C2—C1—Cl1	111.6 (3)	C15—C14—C13	114.9 (3)
C2—C1—H1C	109.3	C15—C14—H14A	108.5
Cl1—C1—H1C	109.3	C13—C14—H14A	108.5
C2—C1—H1D	109.3	C15—C14—H14B	108.5
Cl1—C1—H1D	109.3	C13—C14—H14B	108.5
H1C—C1—H1D	108.0	H14A—C14—H14B	107.5
C3—C2—C1	115.5 (3)	O5—C15—C14	105.4 (3)
C3—C2—H2A	108.4	O5—C15—H15A	110.7
C1—C2—H2A	108.4	C14—C15—H15A	110.7
C3—C2—H2B	108.4	O5—C15—H15B	110.7
C1—C2—H2B	108.4	C14—C15—H15B	110.7
H2A—C2—H2B	107.5	H15A—C15—H15B	108.8
O1—C3—C2	105.3 (3)	O5—C16—C21	125.9 (3)
O1—C3—H3A	110.7	O5—C16—C17	113.5 (3)
C2—C3—H3A	110.7	C21—C16—C17	120.6 (3)
O1—C3—H3B	110.7	C18—C17—O6	126.9 (3)
C2—C3—H3B	110.7	C18—C17—C16	117.2 (3)
H3A—C3—H3B	108.8	O6—C17—C16	115.9 (3)
O1—C4—C5	126.5 (3)	C17—C18—C19	123.3 (3)
O1—C4—C9	113.7 (3)	C17—C18—H18A	118.4
C5—C4—C9	119.8 (3)	C19—C18—H18A	118.4
C4—C5—C6	122.2 (3)	C20—C19—C18	119.2 (3)
C4—C5—H5A	118.9	C20—C19—C23	119.8 (3)
C6—C5—H5A	118.9	C18—C19—C23	120.9 (3)
N1—C6—C7	122.1 (3)	N2—C20—C19	123.2 (3)
N1—C6—C5	119.4 (3)	N2—C20—C21	119.9 (3)
C7—C6—C5	118.5 (3)	C19—C20—C21	116.9 (3)
C6—C7—C8	118.1 (3)	C16—C21—C20	122.7 (3)
C6—C7—C11	120.8 (3)	C16—C21—H21A	118.6
C8—C7—C11	121.0 (3)	C20—C21—H21A	118.6
C9—C8—C7	123.3 (3)	O6—C22—H22A	109.5
C9—C8—H8A	118.3	O6—C22—H22B	109.5
C7—C8—H8A	118.3	H22A—C22—H22B	109.5
C8—C9—O2	126.8 (3)	O6—C22—H22C	109.5
C8—C9—C4	118.0 (3)	H22A—C22—H22C	109.5
O2—C9—C4	115.2 (3)	H22B—C22—H22C	109.5
O2—C10—H10A	109.5	O8—C23—O7	120.9 (3)
O2—C10—H10B	109.5	O8—C23—C19	126.5 (3)
H10A—C10—H10B	109.5	O7—C23—C19	112.6 (3)
O2—C10—H10C	109.5	O7—C24—H24A	109.5
H10A—C10—H10C	109.5	O7—C24—H24B	109.5
H10B—C10—H10C	109.5	H24A—C24—H24B	109.5
O3—C11—O4	121.7 (3)	O7—C24—H24C	109.5
O3—C11—C7	126.1 (3)	H24A—C24—H24C	109.5
O4—C11—C7	112.1 (3)	H24B—C24—H24C	109.5
			
Cl1—C1—C2—C3	71.5 (4)	Cl2—C13—C14—C15	−70.8 (4)
C4—O1—C3—C2	179.4 (3)	C16—O5—C15—C14	177.8 (3)
C1—C2—C3—O1	63.9 (4)	C13—C14—C15—O5	−64.7 (4)
C3—O1—C4—C5	−0.5 (5)	C15—O5—C16—C21	0.9 (5)
C3—O1—C4—C9	179.3 (3)	C15—O5—C16—C17	−179.3 (3)
O1—C4—C5—C6	−179.4 (3)	C22—O6—C17—C18	−6.1 (5)
C9—C4—C5—C6	0.8 (5)	C22—O6—C17—C16	174.4 (3)
C4—C5—C6—N1	−179.4 (3)	O5—C16—C17—C18	−179.7 (3)
C4—C5—C6—C7	−0.4 (5)	C21—C16—C17—C18	0.1 (5)
N1—C6—C7—C8	178.5 (3)	O5—C16—C17—O6	−0.2 (4)
C5—C6—C7—C8	−0.4 (5)	C21—C16—C17—O6	179.6 (3)
N1—C6—C7—C11	−3.0 (5)	O6—C17—C18—C19	−179.8 (3)
C5—C6—C7—C11	178.1 (3)	C16—C17—C18—C19	−0.4 (5)
C6—C7—C8—C9	1.0 (5)	C17—C18—C19—C20	0.4 (5)
C11—C7—C8—C9	−177.5 (3)	C17—C18—C19—C23	178.9 (3)
C7—C8—C9—O2	179.5 (3)	C18—C19—C20—N2	−179.7 (3)
C7—C8—C9—C4	−0.7 (5)	C23—C19—C20—N2	1.8 (5)
C10—O2—C9—C8	4.2 (5)	C18—C19—C20—C21	0.0 (4)
C10—O2—C9—C4	−175.7 (3)	C23—C19—C20—C21	−178.6 (3)
O1—C4—C9—C8	179.9 (3)	O5—C16—C21—C20	−180.0 (3)
C5—C4—C9—C8	−0.2 (5)	C17—C16—C21—C20	0.2 (5)
O1—C4—C9—O2	−0.2 (4)	N2—C20—C21—C16	179.4 (3)
C5—C4—C9—O2	179.6 (3)	C19—C20—C21—C16	−0.2 (5)
C12—O4—C11—O3	−2.5 (5)	C24—O7—C23—O8	1.3 (5)
C12—O4—C11—C7	177.5 (3)	C24—O7—C23—C19	−179.1 (3)
C6—C7—C11—O3	3.5 (5)	C20—C19—C23—O8	−2.1 (5)
C8—C7—C11—O3	−177.9 (3)	C18—C19—C23—O8	179.3 (3)
C6—C7—C11—O4	−176.4 (3)	C20—C19—C23—O7	178.4 (3)
C8—C7—C11—O4	2.1 (4)	C18—C19—C23—O7	−0.2 (4)

### Hydrogen-bond geometry (Å, °)

**Table:**

*D*—H···*A*	*D*—H	H···*A*	*D*···*A*	*D*—H···*A*
N1—H1A···O3	0.86	2.07	2.709 (4)	131
N1—H1B···O8^i^	0.86	2.36	3.155 (4)	154
N2—H2C···O8	0.86	2.09	2.719 (4)	130
N2—H2C···O8^ii^	0.86	2.43	3.216 (4)	152
N2—H2D···O3	0.86	2.31	3.119 (4)	156

Symmetry codes: (i) *x*−1, *y*, *z*; (ii) −*x*+2, −*y*+1, −*z*+2.
